# Bacterial Colonization in Monofilament Sutures of Different Materials Following Periodontal Surgeries: A Microbial Analysis Study

**DOI:** 10.1155/ijod/6008407

**Published:** 2026-10-10

**Authors:** Abdulaziz Alsakr, Lamia Mohammed Alammar, Mohammed Nasser Alasqah, Abdullah Saad Alqahtani, Khalid Gufran

**Affiliations:** ^1^ Department of Preventive Dental Sciences College of Dentistry Prince Sattam bin Abdulaziz University Alkharj 19472 Saudi Arabia psau.edu.sa; ^2^ Postgraduate Student in Periodontics College of Medicine and Dentistry Riyadh Elm University Riyadh 11421 Saudi Arabia riyadh.edu.sa; ^3^ Ministry of Health Specialized Dental Center Riyadh Saudi Arabia moh.gov.sa

**Keywords:** antimicrobial sutures, bacterial colonization, biofilm formation, infection control, monocryl, monofilament suture, periodontal surgery, PTFE

## Abstract

**Objective:**

This study sought to assess and compare the extent of bacterial colonization on various monofilament suture types, including polytetrafluoroethylene (PTFE), polyglycolide‐co‐caprolactone (PGCL Monocryl), triclosan‐coated polydioxanone (Monocryl Plus), and polyamide (nylon). The investigation also aimed to identify predominant bacterial species colonizing these materials and evaluate their potential impact on wound healing and infection control.

**Methods:**

An in vitro experimental design combined with clinical sampling was employed. Sutures were retrieved from patients following periodontal surgery between days 7 and 10. Microbiological culture methods, colony‐forming unit (CFU) quantification, bacterial identification through Vitek2, and scanning electron microscopy (SEM) were utilized to analyze both aerobic and anaerobic bacterial growth. Statistical analysis was performed to compare bacterial adhesion among the different suture types.

**Results:**

The results revealed that PTFE sutures exhibited the lowest bacterial colonization, recording mean aerobic and anaerobic counts of 9.8 × 10^8^ CFU/mL and 8.49 × 10^8^ CFU/mL, respectively, likely due to their smooth and hydrophobic surface characteristics. In contrast, PGCL Monocryl demonstrated the highest levels of colonization, with aerobic and anaerobic counts of 13.3 × 10^8^ and 13.68 × 10^8^ CFU/mL, and harbored bacteria such as *Lactococcus garvieae*, *Staphylococcus warneri*, and *Streptococcus sobrinus*. Monocryl Plus and nylon showed moderate colonization levels, with the triclosan coating on Monocryl Plus reducing but not preventing bacterial attachment.

**Conclusion:**

PTFE demonstrated the lowest numerical bacterial colonization among the evaluated suture materials. However, the differences between the groups were not statistically significant and should therefore be interpreted with caution. Further studies with larger sample sizes are needed to confirm these findings.

**Clinical Relevance:**

The study provides preliminary evidence on bacterial colonization of different monofilament suture materials. These findings may assist clinicians in selecting suture materials, but larger clinical studies are required before definitive recommendations can be made.

## 1. Introduction

Periodontal surgery plays a central role in preserving oral health, restoring function and esthetics, and managing inflammatory and structural conditions. Common surgical procedures include open flap debridement, crown lengthening, coronally advanced flaps, mucogingival interventions such as free gingival grafts (FGGs) and connective tissue grafts (CTGs), as well as guided tissue regeneration (GTR) [1]. Regardless of the technique, healing begins with the formation of a stable blood clot beneath the repositioned flap. This clot serves as the initial scaffold for inflammatory cell migration, angiogenesis, and subsequent tissue remodeling [2].

Sutures are the most widely used devices for wound closure in oral and periodontal surgeries. Their functions extend beyond mechanical stabilization, including clot protection, hemostasis, and minimizing tissue trauma, all of which facilitate first‐intention healing [3, 4]. An ideal suture material should combine tensile strength, knot security, low tissue reactivity, and resistance to bacterial penetration while remaining easy to handle [5, 6].

Sutures differ in resorbability (absorbable vs. nonabsorbable), structure (monofilament vs. multifilament), and material origin (natural vs. synthetic). Natural sutures such as silk demonstrate good handling but are prone to bacterial colonization and tissue reaction [7, 8]. Synthetic absorbable sutures like polyglycolide‐co‐caprolactone (PGCL) or poliglecaprone‐25 (Monocryl) offer predictable degradation profiles, but concerns remain regarding increased bacterial adherence [9, 10]. Nonabsorbable monofilament sutures, such as nylon, polypropylene, and PTFE, exhibit smoother surfaces and lower microbial affinity, though they may compromise handling and knot security [11].

A major concern with sutures in oral surgery is their role as foreign bodies susceptible to microbial colonization and biofilm formation. The complex oral microbiota includes both commensals and opportunistic pathogens, such as *Porphyromonas gingivalis*, *Aggregatibacter actinomycetemcomitans*, *Fusobacterium nucleatum*, and *Streptococcus mutans* [12]. Colonization of sutures by these species may impair wound healing and increase the risk of surgical site infections (SSIs) [4]. Suture macrostructure significantly influences microbial adherence, with multifilament sutures allowing greater bacterial penetration and fluid wicking compared to monofilaments [4].

To counteract bacterial adhesion, antimicrobial‐coated sutures have been developed, including triclosan‐impregnated or chlorhexidine‐coated materials [13]. While these demonstrate some efficacy in reducing colonization, they do not completely prevent bacterial attachment and may be limited by emerging antimicrobial resistance [13]. Recent investigations continue to explore novel biomaterials and surface modifications to enhance the antimicrobial performance and biocompatibility [14, 15].

Evidence from clinical and experimental studies confirms that bacterial colonization varies significantly across suture materials. PTFE sutures are consistently reported to exhibit lower microbial accumulation compared to absorbable alternatives such as PGCL or Monocryl [16, 17]. Conversely, absorbable sutures have been associated with higher bacterial loads and greater biofilm formation [18]. These findings highlight the critical influence of suture choice on infection control and healing outcomes. Ongoing research is directed toward developing advanced sutures with enhanced antimicrobial and mechanical properties to further optimize healing and reduce postoperative complications [19].

However, questions remain about their susceptibility to bacterial colonization in the oral environment, where the microbial load is inherently high. Understanding which bacterial species most commonly adhere to monofilament sutures is crucial as this may directly influence infection rates following surgery. Moreover, the relationship between bacterial adhesion and postoperative complications underscores the need for deeper exploration into how material characteristics contribute to microbial persistence.

This study is guided by the hypothesis that the physicochemical properties of monofilament suture materials, such as their surface smoothness, tensile strength, and the presence or absence of antimicrobial coatings, play a significant role in bacterial colonization. It is postulated that coated sutures may offer superior resistance to microbial attachment when compared to their uncoated counterparts, thereby reducing the risk of infection. By examining bacterial colonization on different suture materials, this study aimed to improve understanding of how material characteristics may influence microbial adhesion in the oral environment.

Thus, the primary objective of this study was to evaluate and compare bacterial colonization levels on monofilament sutures made from various materials, including polytetrafluoroethylene (PTFE), PGCL/Monocryl, polydioxanone with triclosan coating (Monocryl Plus), and polyamide (nylon). The study further aimed to identify the predominant bacterial species colonizing these sutures and to analyze their potential implications for wound healing and infection control. Specifically, it sought to (1) assess the role of suture material composition and coatings on bacterial adhesion; (2) determine whether antimicrobial‐coated sutures provide a measurable advantage; and (3) evaluate bacterial colonization associated with different suture materials and discuss their potential clinical implications.

## 2. Material and Methods

### 2.1. Study Design

This research adopted an in vitro experimental design supported by clinical sample collection to assess the degree of bacterial colonization on various types of monofilament suture material. The study was conducted between October 2024 and October 2025. The ethical approval was obtained from the relevant ethics committee at Riyadh Elm University to ensure compliance with ethical standards on 29 October 2024, with the IRB approval number “FPGRP/2024/813/1091/1068.” The study protocol complied with the Declaration of Helsinki, as revised in Fortaleza in 2013. All participants provided written informed consent after being informed about the study’s objectives and details.

The study aimed to isolate and identify the bacteria adhered to sutures used for periodontal surgeries, monitor their growth patterns, and compare them to each other in terms of bacterial colonization. A combination of laboratory analyses and microbiological culture methods addressed the issues related to these bacterial species and their interactions with the suture materials. This approach was justified considering the fact that periodontal surgeries occurred in a highly microbial environment; thus, these factors had to come into play while understanding the interaction of suture materials with bacteria. In addition, this study incorporated both laboratory and clinical conditions to bolster the results against the challenges posed by the surgical setting.

### 2.2. Study Setting

The study was conducted at the Department of Preventive Dentistry/Periodontics Division and Microbiology Laboratory, College of Dentistry‐Riyadh Elm University. This site possessed all necessary infrastructure; surgical facilities performed periodontal procedures, and microbiological advanced tools isolated and cultured bacteria.

### 2.3. Population and Consent

The study population included patients receiving periodontal surgical procedures requiring suture placement. The provision of such patients entering periodontal surgeries enabled the collection of samples of suture materials from surgical sites to reflect the real picture of bacterial colonization dynamics. Prior to the procedure, patients were explained about the intention of the study and obtained written voluntary informed consent.

#### 2.3.1. Inclusion Criteria

The study included medically healthy ASA I patients (American Society of Anaesthesiologists Physical Status Classification System), patients who have not received any pharmacological or antibiotic therapy in the last 3 months, and those undergoing periodontal surgical procedures such as flap surgery, grafting, or implant placement.

#### 2.3.2. Exclusion Criteria

Patients with systemic diseases or receiving any medication affecting wound healing, pregnant women, smokers, alcoholics, and those with removable or fixed prostheses. These criteria were established to minimize confounding factors, thus allowing bacterial colonization results to mainly originate from suture material properties rather than from any underlying health condition or external treatment. People who met these criteria were excluded from the study.

### 2.4. Sample Size

The sample power analysis was done using G‐Power software v 3.1.9.7. We assumed a priori that 10% of the population would drop out from the study by 7–10 days (*α* = 0.05 and 1−*β* = 0.80). A sample 48 individuals were enrolled in the study, each group composing of 12 individuals.

### 2.5. Data Collection Method

#### 2.5.1. Procedure

All eligible patients underwent periodontal surgery under strict aseptic conditions. The four suture materials were allocated to participants using a simple randomization method. A random allocation sequence was generated by an individual who was not involved in patient recruitment, surgical procedures, or microbiological analysis. Allocation was concealed until the time of surgery, and the assigned suture material was provided to the operating surgeon immediately before placement. All surgical procedures and suture placement were performed by a single experienced periodontist, following a standardized surgical protocol to minimize operator‐related variability.

Four types of sutures used in the study were as follows:

1. Nylon—a nonabsorbable monofilament suture with a smooth surface and minimal bacterial adherence. POLYAMID is a sterile, nonabsorbable monofilament surgical suture made from a copolymer of polyamide 6 and polyamide 6/6.
2. Monocryl “PGCL”—the Monocryl suture is an absorbable monofilament surgical suture that degrades over time and demonstrates resistance to bacterial colonization. It is made from a synthetic copolymer of polyglycolic acid and polycaprolactone, with an empirical molecular formula represented as (C2H202)m (C6H1002)n.
3. Monocryl Plus “P‐dioxanone”—this is an absorbable monofilament suture made from polyester poly (P‐Dioxanone) and coated with triclosan, an antimicrobial agent. The triclosan coating provides an additional layer of protection.
4. PTFE—nonabsorbable monofilament sutures are made from PTFE) polymer. These sutures feature a hydrophobic surface and exhibit high resistance to bacterial colonization.

A simple loop suture was placed interdentally without a Coe‐pack dressing after the periodontal surgery by a single trained surgeon. A suture length after the knot placement was 3–4 mm in all loops. All the patients were instructed to maintain good oral hygiene by rinsing the mouth with salt water twice daily. The amoxicillin was prescribed to all the patients at 500 mg tid for 5 days. None of the patients were prescribed an antibacterial mouthwash.

#### 2.5.2. Suture Removal

The sutures were removed after a period of seven to 10 days. The extraction of the suture materials was performed carefully, adhering to standard procedures in a sterile environment using sterile scissors and tweezers. Immediately upon removal, the sutures were placed into sterile tubes containing reduced transport fluid medium (RTF) to maintain microbial viability. The aerobic samples were transferred into nutrient broth, while the anaerobic samples were placed into thioglycolate broth. Subsequently, the samples were transported to the microbiology laboratory for analysis, where four millimeters of each suture material were examined.

### 2.6. Detection of Microorganisms and Laboratory Techniques

#### 2.6.1. Bacterial Culture

After 24–48‐h incubation at 37°C in a Heraeus incubator, we proceed with subculturing the bacterial isolates. Aerobic bacteria are directly plated onto Columbia blood agar plates (Biolife Manufacturing Company) for incubation. In contrast, anaerobic cultures are placed in an anaerobic jar utilizing Thermo Scientific AnaeroGen 2.5 L bags to create an optimal anaerobic atmosphere. We carefully select colonies with varying morphologies, colors, sizes, and hemolytic reactions to ensure a comprehensive representation of the predominant bacterial types present (Figure 1).

**Figure 1:**
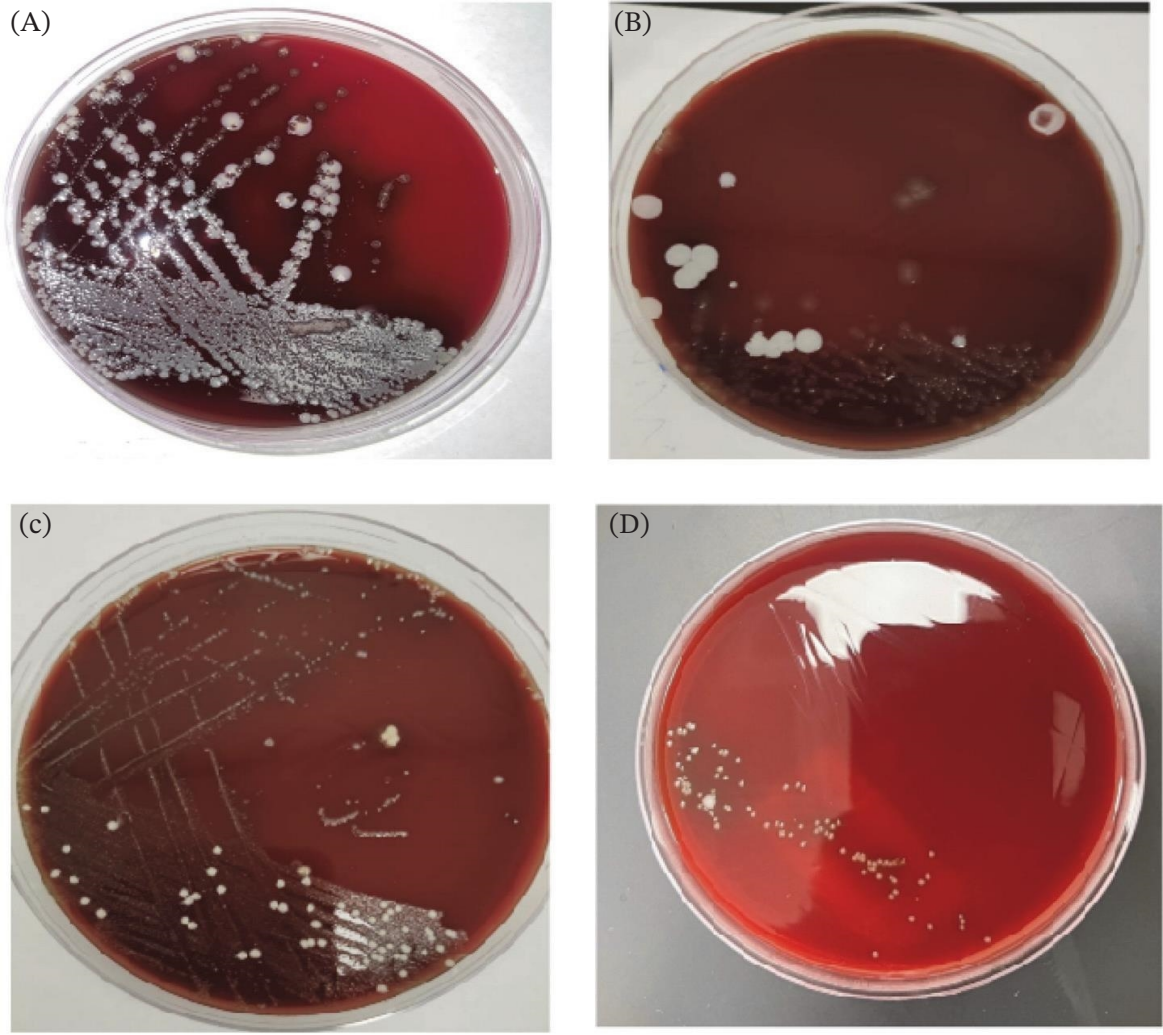
Aerobic and anaerobic bacteria growth on Colombia Blood Agar media. (A) Aerobic bacteria grown around polyamide sutures; (B) anaerobic bacteria grown around polyamide sutures; (C) aerobic bacteria grown around PTFE sutures; (D) anaerobic bacteria grown around PTFE sutures.

#### 2.6.2. Differentiation

Following bacterial incubation, a Gram stain is executed to distinguish between Gram‐positive and Gram‐negative organisms. Morphological characterization involves performing the Gram staining technique, after which cellular morphologies are analyzed using a Motic oil immersion light microscope at a magnification of 1000x.

#### 2.6.3. Colonies‐Forming Unit (CFU)

Colony counting was conducted using a colony counter to determine the number of CFUs. A serial dilution method was employed, utilizing a set of seven tubes, each containing 9 mL of 0.9% saline solution. The bacterial suspension was prepared by thoroughly mixing the organisms with the nutrient broth to ensure a uniform distribution prior to dilution.

To initiate the dilution series, 1 mL of the suspension was aseptically transferred to the first tube using a sterile pipette. Following thorough mixing, 1 mL was aliquoted from the first tube into the second tube, which was then also mixed vigorously. This procedure was replicated by sequentially transferring 1 mL from each tube to the next, continuing until the seventh tube in the series was reached.

The dilutions were as follows:

– Tube 1: 1:10
– Tube 2: 1:100
– Tube 3: 1:1,000
– Tube 4: 1:10,000
– Tube 5: 1:100,000
– Tube 6: 1:1,000,000
– Tube 7: 1:10,000,000

This systematic approach allows for accurate measurements of CFU based on the dilution levels.

#### 2.6.4. Organisms’ Identification

Bacterial identification is performed using the Vitek2 machine. A test tube with microorganism suspension is positioned into a special rack (cassette), and an identification card is inserted into the neighboring slot while the transfer tube is connected to the corresponding suspension tube.

#### 2.6.5. Microbiological Quality Control

All microbiological procedures were performed by trained personnel, following standardized laboratory protocols. Sterile instruments, culture media, and transport media were used throughout the study. Samples were processed under aseptic conditions, and appropriate incubation conditions were maintained for both aerobic and anaerobic cultures to ensure the consistency and reliability of the microbiological findings.

#### 2.6.6. Scanning Electronic Microscopy (SEM)

To examine the surface and structural characteristics of the sutures, samples of all suture materials used in this study were randomly selected and analyzed using SEM. For SEM preparation, the suture materials were initially incubated for 30 min, followed by fixation in 2.5% glutaraldehyde at 4°C for a minimum of 2 h. The samples were subsequently dehydrated through a graded ethanol series ranging from 50% to 100%. Critical‐point drying was then performed using liquid carbon dioxide with a CPD 030 apparatus (Baltec, Wallruf, Germany). Following this, the samples were coated with a gold‐palladium alloy using an SCD 040 sputter coater (Baltec, Wallruf, Germany) for 80 s. The prepared samples were analyzed using a JSM‐7610F Schottky Field Emission SEM operated at an accelerating voltage of 10 kV. The resulting images were digitized for further evaluation. Figure 2 shows the SEM images of the knot region of various sutures at different magnifications (×100, ×5000, and ×10,000) taken seven to 10 days after suture removal.

**Figure 2:**
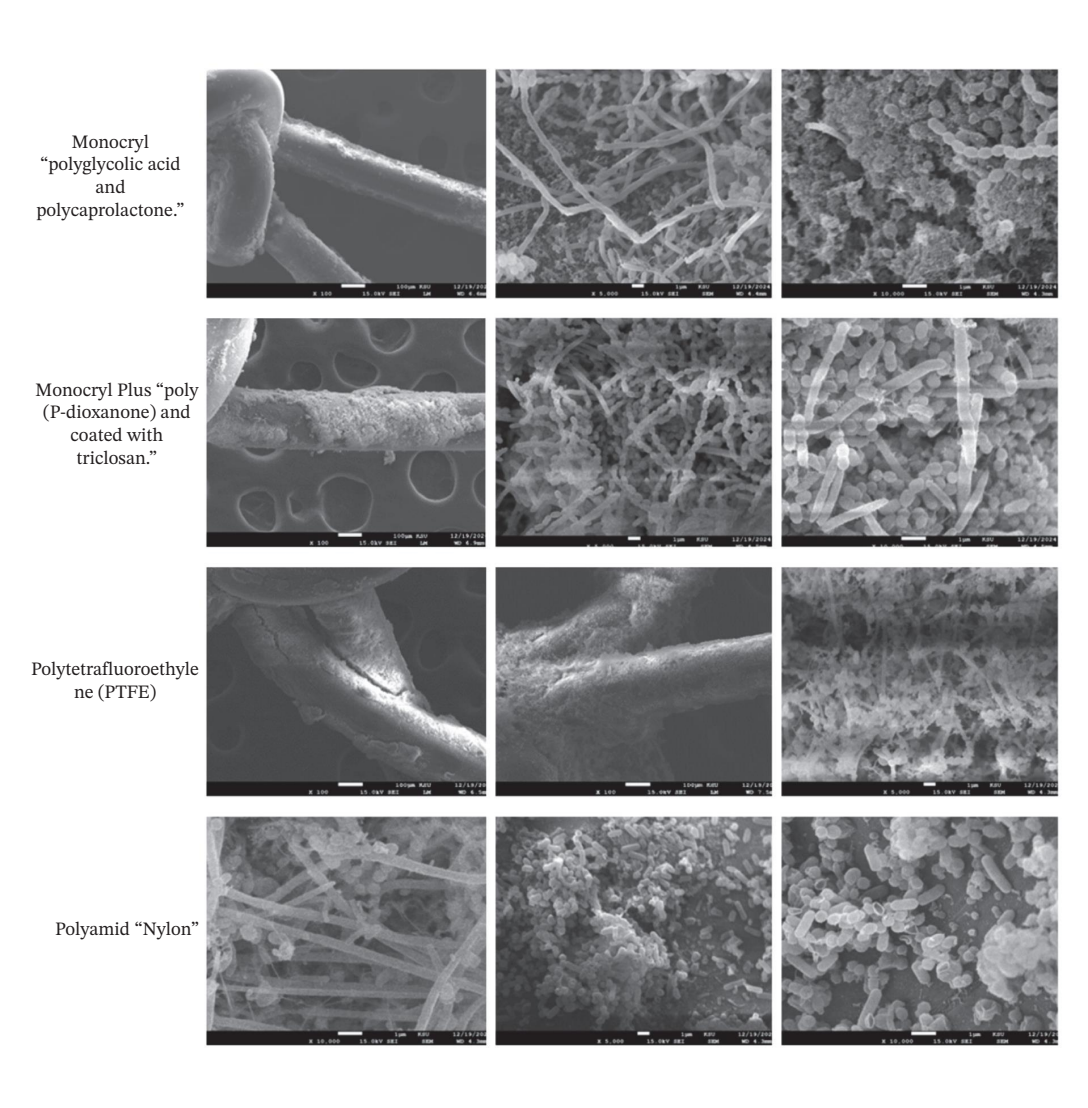
SEM images of knots region of different sutures with different magnifications ×100, ×5000, and ×10,000 after seven to 10 days of suture removal.

### 2.7. Statistical Analysis

The Statistical Package for the Social Sciences (SPSS) 23.0 software package (Chicago, IL, USA) was used for data analysis. The descriptive statistical data were reported in the form of the mean, standard deviation, percentage, and frequency. The chi‐square test was applied for categorical data. The ANOVA test was used to compare the continuous data between the four suture groups. Since the overall ANOVA was not significant, post hoc testing is generally not indicated. A *p*‐value of ≤ 0.05 was considered statistically significant.

### 2.8. Ethical Considerations

The institutional ethics review boards reviewed and approved the study protocol (FPGRP/2024/813/1091/1068). Only approved sutures for clinical use were tested. Patients underwent normal postoperative care and regular follow‐ups to look for any adverse events (File S1).

## 3. Results

The mean aerobic bacteria CFU counts of 9.8, 10.1, 12.5, and 13.3 were recorded in the following suture types: PTFE, P‐Dioxanone “Monocryl Plus,” polyamide “Nylon,” and PGCL “Monocryl”, respectively. The highest growth was observed in PGCL “Monocryl” and the lowest was in PTFE sutures.

The mean anaerobic bacteria CFU counts of 8.49, 13.59, 9.81, and 13.68 were recorded in the following suture types: PTFE, P‐Dioxanone “Monocryl Plus,” polyamide “Nylon,” and PGCL “Monocryl,” respectively. The highest growth was observed in PGCL “Monocryl” and the lowest was in PTFE sutures.

The highest aerobic Gram‐positive bacteria were recorded in the P‐Dioxanone “Monocryl Plus” suture (28.2%) group. Both PTFE and PGCL “Monocryl” showed the same proportion (25.6%) of aerobic Gram‐positive bacteria. The highest aerobic Gram‐negative bacteria were recorded in polyamide “Nylon” (50%) followed by PGCL “Monocryl” (25%). The anaerobic Gram‐positive bacteria were highest in polyamide “Nylon” (28.9%), followed by PTFE (26.3%), while anaerobic Gram‐negative bacteria were highest in P‐Dioxanone “Monocryl Plus” (44.4%) (Figure 3).

**Figure 3:**
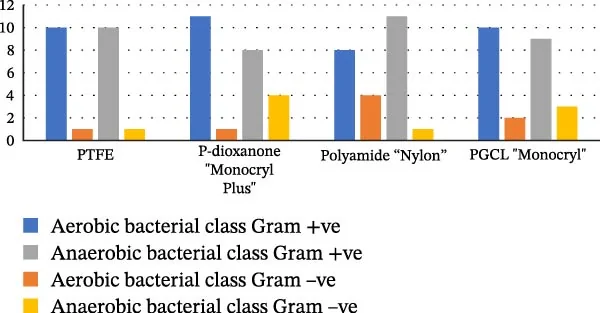
Comparison of aerobic and anaerobic bacterial counts between different suture materials.

The comparison of aerobic bacterial growth between four suture types showed a statistically nonsignificant difference (*p* = 0.48). The highest bacterial growthwas recorded in PGCL “Monocryl” (13.26) and lowest in PTFE (9.8) (Table 1).

**Table 1:** Comparison of mean aerobic bacterial growth between four groups.

Suture materials	Mean	SD	95% CI	*p*‐Value
PTFE	9.80	2.33	8.32–11.28	0.48
P‐Dioxanone “Monocryl Plus”	10.05	3.66	7.72–12.38	
Polyamide “Nylon”	12.55	3.56	10.29–14.81	
PGCL “Monocryl”	13.26	5.62	9.69–16.83	

*Note:* ANOVA test.

The comparison of anaerobic bacterial growth between four suture types showed a statistically nonsignificant difference (*p* = 0.21). The highest bacterial growth was recorded in PGCL “Monocryl” (13.68) and the lowest in PTFE (8.49) (Table 2).

**Table 2:** Comparison of anaerobic bacterial growth between four groups.

Suture materials	Mean	SD	95% CI	*p*‐Value
PTFE	8.49	2.46	6.93–10.05	0.21
P‐Dioxanone “Monocryl Plus”	13.59	5.87	9.86–17.32	
Polyamide “Nylon”	9.81	3.69	7.47–12.15	
PGCL “Monocryl”	13.68	4.69	10.70–16.66	

*Note:* ANOVA test.

In the PTFE suture, the most predominant bacteria were *Streptococcus constellatus* grp (27.3%). In the P‐Dioxanone “Monocryl Plus” suture, *Staphylococcus aureus* (16.7%) and oralis (16.7%) were most predominant. However, in polyamide “Nylon,” *Aeromonas salmonicida* (25%) and *Streptococcus oralis* (25%) were most prevalent. The following bacteria, *Lactococcus garvieae*, *Staphylococcus warneri*, *Streptococcus gordonii*, *Streptococcus sobrinus*, *Streptococcus oralis*, and Staphylococcus epidermis, were most prevalent in PGCL “Monocryl.” The aerobic bacterial growth when compared between two sites, resective surgery and regenerative surgery showed a statistically nonsignificant difference (0.32). The growth in resective surgery was 12.73 and at regenerative surgery was 10.79. The anaerobic bacterial growth when compared between two sites, resective surgery and regenerative surgery, showed a statistically nonsignificant difference (0.42). The growth in resective surgery was 12.65 and at regenerative surgery was 10.81 (Table 3).

**Table 3:** Comparison of anaerobic bacterial growth between the resective and regenerative sites.

Anaerobic bacterial growth		Site	Mean	SD	95% CI	*p*‐Value
Anaerobic bacterial growth “CFUs”		Resective surgery	12.65	2.32	11.18–14.12	0.42
		Regenerative surgery	10.81	3.40	8.65–12.97	

*Note:* Unpaired *t* test.

## 4. Discussion

The present study investigated the bacterial colonization of various suture types, focusing on both aerobic and anaerobic bacterial growth. The findings revealed no significant differences in the bacterial burden across the four suture materials—PTFE, P‐Dioxanone (“Monocryl Plus”), polyamide (“Nylon”), and PGCL “Monocryl.” Among these, PTFE exhibited the least bacterial colonization, while PGCL “Monocryl” demonstrated the highest colonization. These observations align with previous studies examining bacterial adhesion properties on different suture materials.

The relatively large standard deviations observed in some groups indicate considerable interindividual variability in bacterial colonization. This variability may be related to differences in the oral microbiome, surgical site characteristics, host factors, postoperative healing, and individual oral hygiene practices. In addition, administering postoperative systemic antibiotics to all participants may have further contributed to the variability in bacterial counts. These factors may have reduced the ability to detect statistically significant differences among the suture materials despite the observed numerical trends.

PTFE sutures showed the lowest mean aerobic 9.8 × 10^8^ CFU/mL and anaerobic 8.49 × 10^8^ CFU/mL bacterial growth among the tested suture materials, the predominant bacteria was the *Streptococcus constellatus* group (27.3%). This finding can be attributed to the unique molecular structure and surface characteristics of PTFE sutures. PTFE is a nonabsorbable suture material with a smooth, hydrophobic surface that resists bacterial adhesion. A study by Edmiston et al. [20] has demonstrated that hydrophobic suture materials reduce bacterial adherence and biofilm formation due to minimal surface roughness and the absence of absorbable polymer coatings that could serve as a nutrient source for microbial growth.

Additionally, PTFE sutures have been shown to minimize inflammatory responses in tissues, further reducing the likelihood of bacterial colonization. The lower numerical bacterial colonization observed on PTFE sutures suggests that they may be considered in procedures where minimizing bacterial adhesion is desirable. However, further clinical studies are required to determine whether these microbiological findings translate into improved healing or lower infection rates [21, 22].

Conversely, PGCL “Monocryl” sutures exhibited the highest bacterial colonization, with mean aerobic and anaerobic bacterial growth of 13.3 × 10^8^ 8 CFU/mL and 13.68 × 10^8^ CFU/mL, respectively. The following bacteria, *L, garvieae*, *S. warneri*, *S. gordonii*, *S. sobrinus*, *S. oralis*, and Staphylococcus epidermis, were most prevalent. These bacteria are linked to inflammatory responses and, in some cases, the progression of periodontal disease. PGCL is a synthetic, absorbable monofilament suture with a surface that may facilitate bacterial adhesion. Absorbable sutures like PGCL degrade over time, releasing byproducts that can alter the local pH and potentially promote microbial growth [23, 24].

Furthermore, the surface roughness of absorbable sutures can create microenvironments conducive to bacterial colonization and biofilm formation. Similar findings have been reported in a previous study by Obermeier et al. [25], who observed that absorbable sutures like Monocryl “PGCL” tend to exhibit higher bacterial adhesion compared to nonabsorbable materials. The study emphasized that the degradation process of absorbable sutures could provide nutrients that enhance bacterial proliferation. The relatively higher numerical bacterial colonization observed on PGCL sutures suggests that further investigation is warranted. However, because the differences between groups were not statistically significant, no conclusions can be drawn regarding their clinical performance.

P‐Dioxanone ("Monocryl Plus") and polyamide (“Nylon”) sutures demonstrated intermediate levels of bacterial colonization in this study. The mean aerobic bacterial growth for P‐Dioxanone was 10.1 × 10^8^ CFU/mL, with *Staphylococcus aureus* (16.7%) and *S. oralis* (16.7%) being the most predominant. while polyamide showed slightly higher growth at 12.5 × 10^8^ 8 CFU/mL, with *Aeromonas salmonicida* (25%) and *S. oralis* (25%) were most prevalent. Similarly, anaerobic bacterial colonization was higher in P‐Dioxanone 13.59 × 10^8^ CFU/mL, compared to polyamide 9.81 × 10^8^ CFU/mL.

P‐Dioxanone, like PGCL, is an absorbable suture material, and its degradation products may contribute to bacterial colonization. However, the antimicrobial coating on P‐Dioxanone sutures, such as triclosan, has been shown to reduce bacterial adhesion to some extent, as documented by Storch et al. [13]. This antimicrobial property may explain why P‐Dioxanone sutures demonstrated lower bacterial growth compared to PGCL in the current study. Additionally, our study contrasts with some reports suggesting that antimicrobial‐coated Monocryl significantly reduces bacterial adhesion, indicating that antimicrobial coatings alone may not be sufficient to prevent colonization. Triclosan is a phenolic derivative antibacterial agent exhibiting antiseptic and bacteriostatic properties mostly against Gram‐positive bacteria, with reduced efficacy against Gram‐negative bacteria [13]. In a study by Pelz et al. [26], it was demonstrated that triclosan‐coated sutures exhibited greater antimicrobial activity compared to noncoated sutures; however, the difference was not statistically significant.

Polyamide sutures, on the other hand, are nonabsorbable and possess a smoother surface than absorbable sutures, which may account for their relatively lower bacterial colonization. However, Bhingaradia and Gondaliya [27] found that polyamide sutures can still support bacterial adherence due to their hydrophilic nature, which attracts water and, consequently, bacteria. This characteristic makes polyamide less resistant to colonization compared to hydrophobic materials like PTFE. In another study conducted by Mahesh et al. [9], it was demonstrated that both PTFE and polyamide (nylon) sutures exhibited similar levels of bacterial adherence, with aerobic bacteria reaching ~300 × 10^4^ CFUs per suture and anaerobic bacteria at 20 × 10^4^ CFUs per suture.

Despite their susceptibility to bacterial adhesion, polyamide sutures remain a popular choice for surgical procedures due to their tensile strength, flexibility, and biocompatibility. Their intermediate bacterial colonization levels, as observed in this study, suggest that they may be suitable for use in procedures with a moderate risk of infection. However, clinicians should weigh the benefits of polyamide sutures against their potential for bacterial adhesion, particularly in high‐risk surgical environments.

Since all the sutures in this study were monofilament, the differences in bacterial colonization cannot be attributed to the filament structure alone. Instead, variations in surface chemistry, hydrophobicity, and biocompatibility appear to play a key role in bacterial adhesion. This means that even among monofilament sutures, material choice may influence the bacterial load and could potentially affect the risk of postoperative infection. Because all participants received postoperative systemic amoxicillin, bacterial colonization may have been reduced across all groups, potentially diminishing differences attributable solely to the suture material.

The results show that certain bacterial species are more likely to colonize specific suture materials. For instance, *Streptococcus constellatus*, a Gram‐positive bacterium, was the most prevalent in PTFE sutures (27.3%), while *Staphylococcus aureus* and *S. oralis* were dominant in P‐Dioxanone “Monocryl Plus” sutures (16.7% each). In polyamide “Nylon,” *Aeromonas salmonicida* and *S. oralis* were the predominant species, with both accounting for 25% of the bacterial isolates. PGCL “Monocryl” showed a diverse range of bacteria, including *Lactococcus garvieae*, *S*., and *Streptococcus gordonii*, indicating the diverse microbiota that can colonize absorbable sutures.

The presence of these bacteria is clinically significant, as certain species, such as *Staphylococcus aureus*, are known to be pathogenic and are often associated with SSIs. The identification of these bacterial species provides useful microbiological information and may help guide future studies on how different suture materials influence postoperative outcomes.

The findings of this study provide useful information on the bacterial colonization of different suture materials used in periodontal surgery. Although PTFE showed lower bacterial colonization than the other materials, the differences were not statistically significant. Therefore, these findings should be interpreted with caution and confirmed in larger studies that also evaluate wound healing and other clinical outcomes.

In contrast, the high bacterial colonization associated with PGCL “Monocryl” sutures suggests that their use should be carefully evaluated, particularly in contaminated or high‐risk surgical sites. While absorbable sutures like PGCL are advantageous in certain procedures due to their biodegradability. However, their susceptibility to bacterial colonization may increase the risk of SSIs. Suture physical and chemical properties and SSI risk are still disputed [16]. Clinicians may need to consider alternative materials, such as antimicrobial‐coated sutures or nonabsorbable options, in such cases [28–31].

The sutures in the present study were removed between 7 and 10 days after surgery, consistent with the routine postoperative follow‐up protocol used at our institution. However, the duration of suture retention is an important factor influencing bacterial colonization. Previous studies have reported that bacterial adhesion begins within the first few days after surgery and may increase with prolonged retention. Therefore, the 7–10‐day retention period used in the present study may have contributed to greater bacterial accumulation and may limit direct comparison with studies in which sutures were removed earlier (e.g., 3–7 days). Future studies should standardize suture removal intervals to better evaluate the influence of retention time on bacterial colonization [32].

Although PTFE demonstrated the lowest numerical bacterial colonization in the present study, the differences between the suture materials were not statistically significant. Therefore, no recommendation regarding the superiority of any specific suture material can be made based on the present findings alone. Larger randomized clinical studies evaluating both microbiological and clinical outcomes are required before definitive recommendations can be made. The selection of the suture material should consider its mechanical characteristics, potential for bacterial adhesion, and the clinical requirements of the individual patient and procedure.

This study offers valuable insights into bacterial adherence to various suture materials; however, it has several limitations that should be addressed. First, the study was conducted at a single center with a relatively small sample size, which may have limited the statistical power and the generalizability of the results. Second, all participants received postoperative systemic amoxicillin as part of the standard treatment protocol. Although this standardized postoperative care reduced variability among participants, it may have lowered the overall bacterial burden and reduced the differences in bacterial colonization between the suture materials. Third, bacterial identification relied on conventional culture methods and Vitek2 identification. While these techniques detect viable microorganisms, they do not fully represent the diversity of the oral microbiome because many oral bacteria are difficult to culture. Molecular techniques such as quantitative PCR and 16S rRNA sequencing provide substantially greater sensitivity and allow the comprehensive characterization of bacterial communities. A recent work by Rhoads et al. [33] demonstrated the advantages of integrating molecular microbiological methods with clinical wound healing assessment. Future studies should combine conventional microbiology with molecular approaches to provide a more comprehensive evaluation of bacterial colonization. In addition, plaque index, gingival inflammation, and patient compliance with oral hygiene instructions were not recorded and may have influenced bacterial colonization.

Finally, this study focused only on bacterial colonization and did not evaluate clinical outcomes such as wound healing, postoperative infection, pain, bleeding, or wound dehiscence. Therefore, no conclusions can be drawn regarding the clinical superiority of any suture material. Future multicenter studies with larger sample sizes incorporating molecular microbiological techniques and clinical outcome measures are needed to validate these findings. Additionally, the response of tissues to the materials and the interaction between the host and both adherent and desorbed bacteria are critical areas that deserve further investigation.

Future multicenter randomized clinical trials with larger sample sizes are needed to validate these findings. Such studies should use molecular techniques, such as PCR and 16S rRNA sequencing, along with conventional culture methods, to better characterize bacterial communities. They should also evaluate clinical outcomes, including wound healing and postoperative infection, investigate the interaction between host tissues and bacteria, and assess antibiotic susceptibility to provide a more comprehensive understanding of bacterial colonization of suture materials.

## 5. Conclusion

Within the limitations of this study, PTFE exhibited the lowest numerical bacterial colonization, whereas PGCL Monocryl showed the highest among the evaluated suture materials. However, these differences were not statistically significant and should therefore be interpreted cautiously. Although antimicrobial‐coated sutures exhibited lower bacterial colonization than PGCL Monocryl, bacterial adhesion was not completely prevented. Further, well‐designed randomized clinical trials incorporating both microbiological and clinical outcome measures are required to determine whether differences in bacterial colonization translate into improved postoperative outcomes.

## Author Contributions

Abdulaziz Alsakr and Lamia Mohammed Alammar contributed to conception, design, data acquisition, and interpretation and drafted the manuscript. Mohammed Nasser Alasqah contributed to conception, design, interpretation, and data analysis. Abdullah Saad Alqahtani and Khalid Gufran contributed to interpretation, design, and drafted and critically revised the manuscript.

## Funding

This research received no external funding.

## Disclosure

All authors reviewed the final version and agreed to be accountable for all aspects of the work.

## Ethics Statement

The institutional review board of Riyadh Elm University approved the study protocol (FPGRP/2024/813/1091/1068) dated 29‐10‐2024. Informed consent was obtained from all subjects involved in the study.

## Consent

All the participants gave consent for the publication of the data.

## Conflicts of Interest

The authors declare no conflict of interest.

## Supporting Information

Additional supporting information can be found online in the Supporting Information section.

## Supporting information


**Supporting Information** File S1: This study was reported in accordance with the Checklist for Reporting In Vitro Studies (CRIS) guidelines. The completed checklist is provided in Supporting Information section.

## Data Availability

The datasets used and/or analyzed during the current study are available from the corresponding author upon reasonable request.

## References

[1] Shah R. , Domah F. , Shah N. , and Domah J. , Surgical Wound Healing in the Oral Cavity: A Review, Dental Update. (2020) 47, no. 2, 135–143, 10.12968/denu.2020.47.2.135.

[2] Zhao H. , Hu J. , and Zhao L. , The Effect of Low-Level Laser Therapy as an Adjunct to Periodontal Surgery in the Management of Postoperative Pain and Wound Healing: A Systematic Review and Meta-Analysis, Lasers in Medical Science. (2021) 36, no. 1, 175–187, 10.1007/s10103-020-03072-5. 32613416

[3] Byrne M. and Aly A. , The Surgical Suture, Aesthetic Surgery Journal. (2019) 39, no. Supplement_2, S67–S72, 10.1093/asj/sjz036. 30869751

[4] Faris A. , Khalid L. , and Hashim M. , et al.Characteristics of Suture Materials Used in Oral Surgery: Systematic Review, International Dental Journal. (2022) 72, no. 3, 278–287, 10.1016/j.identj.2022.02.005. PMC9275112 35305815

[5] Kuzu T. E. , Comparison Tensile Strength of Different Sutur Materials, Cumhuriyet Dental Journal. (2022) 24, no. 4, 355–360, 10.7126/cumudj.978167.

[6] Manfredini M. , Ferrario S. , Beretta P. , Farronato D. , and Poli P. P. , Evaluation of Breaking Force of Different Suture Materials Used in Dentistry: An In Vitro Mechanical Comparison, Materials. (2022) 15, no. 3, 10.3390/ma15031082, 1082. PMC8840186 35161027

[7] Dragović M. , Pejović M. , and Stepić J. , et al.Microbial Adherence Affinity and Clinical Characteristics of Polypropylene Versus Silk Sutures in Oral Surgery, Srpski Arhiv za Celokupno Lekarstvo. (2018) 146, no. 5-6, 258–263, 10.2298/SARH170428184D.

[8] Dragovic M. , Pejovic M. , and Stepic J. , et al.Comparison of Four Different Suture Materials in Respect to Oral Wound Healing, Microbial Colonization, Tissue Reaction and Clinical Features—Randomized Clinical Study, Clinical Oral Investigations. (2020) 24, no. 4, 1527–1541, 10.1007/s00784-019-03034-4. 31342245

[9] Mahesh L. , Kumar V. R. , and Jain A. , et al.Bacterial Adherence Around Sutures of Different Material at Grafted Site: A Microbiological Analysis, Materials. (2019) 12, no. 18, 10.3390/ma12182848, 2848. PMC6765980 31487852

[10] Sailakshmi M. , Misra S. , Hs S. , Acharya S. , Moharana A. K. , and Ts D. , Clinical Equivalence of Monoglyde® and Monocryl® Absorbable Poliglecaprone-25 Sutures: A Single-Blind, Randomized Study, Cureus. (2023) 15, no. 5, 10.7759/cureus.38938. PMC10257932 37309337

[11] Shakiba M. , Rezvani Ghomi E. , and Khosravi F. , et al.Nylon—A Material Introduction and Overview for Biomedical Applications, Polymers for Advanced Technologies. (2021) 32, no. 9, 3368–3383, 10.1002/pat.5372.

[12] Lamont R. J. , Koo H. , and Hajishengallis G. , The Oral Microbiota: Dynamic Communities and Host interactions, Nature Reviews Microbiology. (2018) 16, 745–759. 10.1038/s41579-018-0089-x PMC6278837 30301974

[13] Storch M. L. , Rothenburger S. J. , and Jacinto G. , Experimental Efficacy Study of Coated VICRYL Plus Antibacterial Suture in Guinea Pigs Challenged With *Staphylococcus aureus* , Surgical Infections. (2004) 5, no. 3, 281–288, 10.1089/sur.2004.5.281. 15684799

[14] Reinbold J. , Uhde A. K. , and Müller I. , et al.Preventing Surgical Site Infections Using a Natural, Biodegradable, Antibacterial Coating on Surgical Sutures, Molecules. (2017) 22, no. 9, 10.3390/molecules22091570, 1570. PMC6151728 28925959

[15] Makrygiannis I. H. , Nikolaidis A. K. , and Tilaveridis I. , et al.Coated Sutures for Use in Oral Surgery: A Comprehensive Review, Clinical Oral Investigations. (2025) 29, no. 2, 10.1007/s00784-025-06176-w, 109. PMC11794639 39903293

[16] Asher R. , Chacartchi T. , Tandlich M. , Shapira L. , and Polak D. , Microbial Accumulation on Different Suture Materials Following Oral Surgery: A Randomized Controlled Study, Clinical Oral Investigations. (2019) 23, no. 2, 559–565, 10.1007/s00784-018-2476-0. 29717362

[17] Naghsh N. , Yaghini J. , Arab A. , and Soltani S. , Comparison of the Number of Bacterial Colonies Among Four Types of Suture Threads Using Simple Loop Method Following Periodontal Surgery in Patients With Periodontitis: A Single-Blind Randomized Clinical Trial, Dental Research Journal. (2023) 20, no. 1, 10.4103/1735-3327.379628, 71. PMC10361267 37483897

[18] Nadafpour N. , Montazeri M. , and Moradi M. , et al.Bacterial Colonization on Different Suture Materials Used in Oral Implantology: A Randomized Clinical Trial, Frontiers in Dentistry. (2021) 18, 10.18502/fid.v18i25.6935, 25. PMC9355897 35965706

[19] Narasimhan A. K. , Rahul T. S. , and Krishnan S. , Revisiting the Properties of Suture Materials: An Overview, Advanced Technologies and Polymer Materials for Surgical Sutures, 2023, Elsevier, 199–235, 10.1016/B978-0-12-819750-9.00011-5.

[20] Edmiston C. E. , Seabrook G. R. , and Goheen M. P. , et al.Bacterial Adherence to Surgical Sutures: Can Antibacterial-Coated Sutures Reduce the Risk of Microbial Contamination?, Journal of the American College of Surgeons. (2006) 203, no. 4, 481–489, 10.1016/j.jamcollsurg.2006.06.026. 17000391

[21] Parrini S. , Bovicelli A. , and Chisci G. , Microbiological Retention on PTFE Versus Silk Suture: A Quantitative Pilot Study in Third Molar Surgery, Antibiotics. (2023) 12, no. 3, 10.3390/antibiotics12030562, 562. PMC10044079 36978429

[22] Polykandriotis E. , Ruppe F. , and Niederkorn M. , et al.Polytetrafluoroethylene (PTFE) Suture Vs Fiberwire and Polypropylene in Flexor Tendon Repair, Archives of Orthopaedic and Trauma Surgery. (2021) 141, no. 9, 1609–1614, 10.1007/s00402-021-03899-9. PMC8354926 33876291

[23] Otten J. E. , Wiedmann-Al-Ahmad M. , and Jahnke H. , et al.Bacterial Colonization on Different Suture Materials--A Potential Risk for Intraoral Dentoalveolar Surgery, Journal of Biomedical Materials Research Part B: Applied Biomaterials. (2005) 74, no. 1, 627–635, 10.1002/jbm.b.30250. 15889436

[24] Krishna S. , Bhaskaran R. , Kumar S. P. , Krishnan M. , and Lakshmanan S. , Microbiological Evaluation of Four Different Suture Materials Used for the Surgical Removal of Impacted Lower Third Molars: A Single-Center Prospective Comparative Study, Cureus. (2023) 15, no. 11, 10.7759/cureus.49370. PMC10749685 38146549

[25] Obermeier A. , Schneider J. , and Harrasser N. , et al.Viable Adhered *Staphylococcus aureus* Highly Reduced on Novel Antimicrobial Sutures Using Chlorhexidine and Octenidine to Avoid Surgical Site Infection (SSI), PLoS ONE. (2018) 13, no. 1, 10.1371/journal.pone.0190912. PMC5760023 29315313

[26] Pelz K. , Todtmann N. , and Otten J. E. , Comparison of Antibacterial-Coated and Non-Coated Suture Material in Intraoral Surgery by Isolation of Adherent Bacteria, Annals of Agricultural and Environmental Medicine. (2015) 22, no. 3, 551–555, 10.5604/12321966.1167733. 26403133

[27] Bhingaradia Y. M. and Gondaliya S. J. , Utilization of Nonabsorbable (Polyamide Black) Buried Subcutaneous Suture Material in Closure of Elliptical Excision in Various Indications for Better Aesthetic Outcome, Journal of Cutaneous and Aesthetic Surgery. (2021) 14, no. 2, 215–219, 10.4103/JCAS.JCAS_93_19. PMC8423201 34566365

[28] Ercan U. K. , İbiş F. , and Dikyol C. , et al.Prevention of Bacterial Colonization on Non-Thermal Atmospheric Plasma Treated Surgical Sutures for Control and Prevention of Surgical Site Infections, PLoS ONE. (2018) 13, no. 9, 10.1371/journal.pone.0202703. PMC6124751 30183745

[29] Yang Y. , Zhou Z. , and Ma R. , et al.Antimicrobial-Coated Sutures Versus Non-Coated Sutures in Reducing Surgical Site Infection: An Updated Systematic Review and Meta-Analysis, Journal of Hospital Infection. (2024) 150, 40–50, 10.1016/j.jhin.2024.04.027. 38823643

[30] Daood U. , Ilyas M. S. , and Ashraf M. , et al.A Novel Coated Suture Displays Antimicrobial Activity Without Compromising Structural Properties, Journal of Oral and Maxillofacial Surgery. (2024) 82, no. 9, 1147–1162, 10.1016/j.joms.2024.05.004. 38830601

[31] Schmitz N.-D. , Ovington L. , Berlin J. , Zhang S. , and Collier J. , Optimal usage of Antibacterial Sutures for Wound Closure in Clinical Trials Addressing SSI, The Lancet. (2023) 401, no. 10387, 1497–1498, 10.1016/S0140-6736(23)00668-2. 37149304

[32] Sala-Pérez S. , López-Ramírez M. , and Quinteros-Borgarello M. , et al.Antibacterial Suture Vs Silk for the Surgical Removal of Impacted Lower Third Molars. A Randomized Clinical Study, Medicina Oral, Patologia Oral y Cirugia Bucal. (2015) 21, no. 1. 10.4317/medoral.20721 PMC4765748 26615503

[33] Rhoads D. D. , Wolcott R. D. , Sun Y. , and Dowd S. E. , Comparison of Culture and Molecular Identification of Bacteria in Chronic Wounds, International Journal of Molecular Sciences. (2012) 13, no. 3, 2535–2550, 10.3390/ijms13032535. PMC3317672 22489109

