# Supplementary material for: Bacterial Colonization in Monofilament Sutures of Different Materials Following Periodontal Surgeries: A Microbial Analysis Study
Source: Int J Dent. 2026 Oct 10;2026:6008407. doi: 10.1155/ijod/6008407 (PMC13653258; doi:10.1155/ijod/6008407)
Supplement: Supplementary file 1 [file IJOD-2026-6008407-s001.pdf]

CRIS Guidelines (Checklist for Reporting *In-vitro* Studies)*

Section/Topic	Item No	Checklist item	Reported on page N
**Title and abstract**			
	1a	Identification as an in vitro/laboratory study in the title	1 1
	1b	Structured summary of trial design, methods, results, and conclusions	1 2-3
**Introduction**			
Background and objectives	2a	Scientific background and explanation of rationale	2-3 3-6
	2b	Specific objectives or hypotheses	3 6
**Methods**			
Interventions	3	The intervention for each group, including how and when they were actually administered, with sufficient detail to allow replication	5 6
Outcomes	4	Completely defined pre-specified primary and secondary outcome measures, including how and when they were assessed	4-5 6-12
Sample size	5	How sample size was determined	4 8
Randomisation:			
Sequence generation	6	Method used to generate the random allocation sequence	NA NA
Allocation concealment mechanism	7	Mechanism used to implement the random allocation sequence (such as sequentially numbered containers), describing any steps taken to conceal the sequence until interventions were assigned	NA NA
Implementation	8	Who generated the random allocation sequence, who enrolled teeth, and who assigned teeth to intervention	NA NA
Blinding	9	If done, who was blinded after assignment to interventions (for example, care providers, those assessing outcomes) and how	4 (those assessing outcome)
Statistical methods	10	Statistical methods used to compare groups for primary and secondary outcomes	6 12
**Results**			
Numbers analysed	11a	For each group, number of 'items' (drugs) included in each analysis and whether the analysis was by original assigned groups	7-8, Table1 16 (Table 1)

Outcomes and estimation	11b	For each primary and secondary outcome, results for each group, and the estimated effect size and its precision (such as 95% confidence interval)	7-8, 17 (Table 2 19 (Table 3 Table 2-3
**Discussion**			
Limitations	12a	Trial limitations, addressing sources of potential bias, imprecision, and, if relevant, multiplicity of analyses	11 21
Generalisability	12b	Generalisability (external validity, applicability) of the trial findings	10-11 21
Interpretation	12c	Interpretation consistent with results, balancing benefits and harms, and considering other relevant evidence	9-11 15-21
**Other information**			
Protocol	24	Where the full trial protocol can be accessed, if available	NA NA
Funding	25	Sources of funding and other support (such as supply of drugs), role of funders	Title page

*This checklist was developed by the authors based on the following sources:

1. Krithikadatta J, Gopikrishna V, Datta M. CRIS Guidelines (Checklist for Reporting In-vitro Studies): A concept note on the need for standardiz guidelines for improving quality and transparency in reporting in-vitro studies in experimental dental research. *J Conserv Dent.* **2014**;17(4):301–304.
2. Faggion CM Jr. Guidelines for reporting pre-clinical in vitro studies on dental materials. *J Evid Based Dent Pract.* **2012**;12(4):182–189.
3. www.consort-statement.org.
